# Supplementary material for: In Silico Identification and Characterization of Satellite DNAs in 23 Drosophila Species from the Montium Group
Source: Genes (Basel). 2023 Jan 23;14(2):300. doi: 10.3390/genes14020300 (PMC9957191; doi:10.3390/genes14020300)
Supplement: Supplementary file 1 [file genes-14-00300-s001.zip › File S1, Figures S1 and 2, Tables 2 and 3.pdf]

File S1. Satellite DNA consensus sequences of 101 satellite DNAs identified in species from the *montium* group.

>dmgsat-1_D.asahinai
GAAAATACACGGCTTTTGGCTATCACTTCCTTTTTGGCTATAAGAGGCTCTTAAGAAGACCTTACTTTCTCACATTTTTTGAATCAAAAAATGTTTCGAAAAATATGATTTTTT
TTTTAATAATCTAGTATAAAAACCTTCAAAACTATGCCAAAACAGCCTTAATTTCAATTTGGAAAGCTTTTCTGTGTCAGTTTTTATTAGATTAATAAATCTGCTTATTAAGAT
TAGAAATTTAAACAAATAATTTTTTTGCGATGTTTTGAAAAAACTAAGTTTCCCCCTTATCAAATTTTGAAAAATCGATAAAAAATTGTTTTCTGATATTTTTCAATATTTTGG
TTTTAAGTGTTTACAATTTTAACGACGAATCG
>dmgsat-1_D.rufa
GAAAATACACGGCTTTTGGCTATCACTTCCTTTTTGGCTATAAGAGGCTCTTAAGAAGACCGTACTTTTGATTCAAAAAATGTGTCGAAAAATGTTTTTTTTTTTTTTGGGAA
ATCTAAGATAAAAACCTTCATAACTAAGCCAAAACAGCCTTAATTTCAATTCGGAAAGCTTTTCTGTGTCAGTTTTTATTAGATTAATAAATCTGCTTATTAAGATTAGAAATT
TAAGCAAATAAATTTTTCGTGATTTTTTGAAAAAATACTGCCATCCCCCCTTATCACATTTTGAAAAATCGATAAAAAAAATTTTTCTGATATTTTTCAATATTTTGGTTTTAA
GTGTTTACAATTTTAACGACGAATCG
>dmgsat-1_D.lacteicornis
GAACATACACGGCTTTTGGCTATCACTTCCTTTTTGGCTATAAGAGGCTCTTAGGAAGATTTTACTTTTCCACATTTTTTGAATCAAAAAATGTTTCGAAAAATATGATTTTTT
TTTTAATAATCTAGGATAAAAACCTTCAAAACTATGCCAAAACAGCCTTAATTTCAATTCGGAAAGCTTTTCTGTGTCAGTTTTTATTAGATTAATAAATCTGCTTATTAAGAT
TAGAAATTTAAACAAATAATTTTTTTGCGATTTTTTGAAAAAACTAAGTTTCCCCCTTATCAAATTTTGAAAAATCGATAAAAAATTGTTTTCTGATATTTTTCAATATTTTGG
TTTTAAGTGTTTACAATTTTAACGACGAATCG
>dmgsat-1_D.tani
GAAAATACACGGCTTTTGGCTATCACTCGCTTTTTGGCTATAAGAGGCTCTTAAGAAGACCTCAAATTTTCACATTTTTGGGTTAAAAATAAAATTCCAAATATATGATTTTTT
TTGAAAAATCTAAGATAAAAATTACCATAACTAAGTCAAAACAGCCTTAAATTCAATTCGGGAAGCTGTTCTATGTTAGATTTTATTAGCTTATAAATTCTGCTTATTTAGTTT
AGAATTCTTGACAATTAAATTTTTTGCCAATTTTCGACATTTTTAATGATGAAAGTTATGAAAATTTTGAAAAATCGATCAAAATTTTTTTTCTGGAATTTTTTAATATTTTGG
CTTTAAGTGTTTACAATTTTAACGACGAATCC
>dmgsat-1_D.auraria
GAAAATACACGCCTTTTTGCTATCACTCCCTTTTTGCCTATAGGAGGAGCTGAAGAAGACCTTAAATTTTCAAAATATTTTTATTTTTTAAATATGATAATTTTTTGAAAAAAA
CTTAGACAAAAATAGTCATAACTAAGCTAAAACAGCCTTAATTTCAATTTGGAAAGCTGTTCTATGTTAGGTTTTATGAAAATAATAAATCTGCATACTCAGTTTAAAAATTTA
AACAAATAAATTTTATGCCAATTTTTGAACATTTTAACGACCCCCGACCTTCTAACATTTTAAAAAATCAAAAAAAAAATTTTTTCTGATATTTTTCAATATTTTGGTTTTAAA
TGTTTGTAATTTTAATGACGAATCC
>dmgsat-1_D.triauraria
GAAAATACACGCCTTTTTGCTATCACTCCCTTTTTGCCTATAGGAGGAGCTGAAGAAGACCTTAAATTTTCAAAATATTTTTATTTTTTAAATATGATAATTTTTTGAAAAAAA
CTTAGACAAAAATAGTCATAACTAAGCTAAAACAGCCTTAATTTCAATTTGGAAAGCTGTTCTATGTTAGGTTTTATGAAAATAATAAATCTGCATACTCAGTTTAAAAATTTA
AACAAATAAATTTTATGCCAATTTTTGAACATTTTAACGACCCCCGACCTTCTAACATTTTAAAAAATCAAAAAAAAAAATTTTCTGATATTTTTCAATATTTTGGTTTTAAAT
GTTTGTAATTTTAATGACGAATCC
>dmgsat-2_D.asahinai
TTCTGCCCATAACTTCTAAACGACTTAAGCTACAGAAATGATGTAAGTACTGTTGTCTTCTGGCGTTAAATACGCAGCCAACGACACCACATTCATCCCGATCGGATGCTCCGT
TGAAAAGATATTCAATAAAGATGATTTTACCGTTGTTTTTTTTGTGTTA
>dmgsat-2_D.rufa
TTCTGCCCATAACTTCTAAACGACTTAAGCTACAGAAATGATGTAAGTACTGTTGTCTTCTGGCGTTAAATACGCAGCCAACGATACCACATTCATCCCGATCTGATGCTCCGT
TGAAAAGATATTCAATAAAGATGATTTTACCGTTGTTTTTTTTGTGTTA
>dmgsat-2_D.lacteicornis
TTCTGCCCATAACTTCTAAACGACTTAAGCTACAGAAATGATGTAAGTACTGTTGTCTTCTGGCGTTAAATACGCAGCCAACGACACCACATTCATCCCGATCGGATGCTCCGT
TGAAAAGATATTCAATAAAGATGATTTTACCGTGGTTTTTTTTGTGTTA
>dmgsat-2_D.tani
TTCTGCCCATAACTTCTAAACGGCTTAAGCTACAGAAATAATGTAAGTACTGTTGTCTTCTGCCGTTAAATACGCAGCCAACGACACCACATTCATCCCGATCTGATGCTCCGT
TGAAAAGATATTCAATAAAGAATAGATATAGATAGTTAAAAAAATATGATTTTTTCGATGTTTTTTTTGTGTTA

>dmgsat-2_D.auraria
TTCTGCCCATAACTTCTAAACGGCTTAAGTTACAGAAACAGTTTAAATGTTGTTTTGCTCTAGTTTTGAGTACGCGTCGAACAATACCTCAATCATCCCGATCAGATACTCTGT
TAAAAAAAATACTCAACAAAGAAGTTTTTACAGTTTAATTTTTTTTAATTAC
>dmgsat-2_D.triauraria
TTCTGCCCATAACTTCTAAACGGCTTAAGTTACAGAAACAGTTTAAATGTTGTTTTGCTCTAGTTTTGAGTACGCGTCGAACAATACCTCAATCATCTCGATCAGATACTCTGT
TGAAAAGATATTCAACAAAGAAGTTTTTACAGTTTAATTTTTTTTAATTAC
>dmgsat-3_D.asahinai
TTTTTTTCATGCATGAA
>dmgsat-3_D.rufa
TTTTTTTCATGCATGAA
>dmgsat-3_D.lacteicornis
TTTTTTTCATCGATGAA
>dmgsat-4_D.punjabiensis
AAAAATATATCAAAATATAT
>dmgsat-4_D.watanabei
AAAAATATATAAAAACATGC
>dmgsat-5_D.mayri
TTTTTAGAGTTTCTGGATGGAATTTTTGAGTTTTAAGGGGTTTTAAGGGGGCAAACGTTTGTTATTTTGTTTTAAGAGTTATTTGATGAAATTGTTTAGTTTTAAGGGGTGGGC
ATTTTAGATTTTGTAGTTTTAAGAGATGGGCAGAAGTTTTTTTCTTAAATTTTTTAGTTTTAAGGATTTTTTTTAT
>dmgsat-5_D.birchii
TTTTTAGTATTTCTCGATGAAAATGTTTAGTTTTAAAGGGTGGGCATTTTATATTTTGTAGTTTTAAGAGATGGGCAGAAGTTTTTTTTTTTAATTTTTTAGTTTTAAGGATTT
TTTTTAT
>dmgsat-6_D.serrata
GTCAGAAGTGTCAGTTTCAGATGTGTCAGT
>dmgsat-6_D.bunnanda
GACACTTCTGTCACTGACACTTTTGGCACTGACACTTCTGACACTGACACTTTCACTACTGACACT
>dmgsat-7_D.mayri
ATATTATTCCTGAATTTCGGTTTTAATTTCATGAAAATCGGCCTACTATATCCTATAGCTTCCATAGGAACAATCAGGAAAATAAAAGAAAACCCCATAATTTTGTCAGTTTTC
AACATATTTTCATCAAAGTTGAGATTTAGCCGTTTT
>dmgsat-7_D.serrata
ATATTACTCTACAATTTTGTGTAAAATTTTATGAAAATCGGACTACTATATCATATAGCTCCCATAGGAACGATCGAGAAATTAATACAATTAAAAATTATAACTTTTTCAGTT
TTCAACAGAATTTCATCAAATTTGAGATATAGCCATTTT
>dmgsat-8_variant_1_D.truncata
AACATGAAAATCGAAGTTTGGGAATGCCATAACTTGGCCAAAAATCCATCAATTTCAATTCTGGAAGCACAGAAGTGTTTGTTTTTATTAGCTTAACAACACTGCAAACCAAAT
TTATTAAATTTCCATAGTTTCAGATTTTTCGAATTTTTCACAATTTTGACGACCCCCACCCTTATCTTTTTTTGGAATAATGGC
>dmgsat-8_variant_2_D.truncata
AACAAAAAAATCGAAGTTGGGAATACCATAAATTGGCCAAAAATCTATCAATTTTAAATCTGAGAGCACAACAGTATTATTTTTATTAGCTTAACAAACCAGCAAACCCAATTA
AATAATTTTCATTAATATCAAAGATTTTGAATTTTTCACAATTTTCACGACCCCTTATCATTTTCGAAAAAAAAAACATC
>dmgsat-8_D.birchii
ATCATGAAAATCAAAATTTTGAAATGCCATAACTTGGCTTTAAATAAACTAATTTTAATTCTGTAAGCACAGAAGTGCTTGTTTTTGTTAGCTCTACAAAGCTGCAAACCTAAA
TTTATCAATTTATTTAAATTAAAAAATTTGAATTTTTTAATGGGGTACCCCCTTATCTTAAAAAATAA
>dmgsat-9_D.birchii
GAATAAGAATAAGAATAAGAATAA
>dmgsat-9_D.bunnanda

GAATAAGAATAAGAATAAGAATAA
>dmgsat-10_D.bocki
AAAAAAGAAGGAGTAGAAGACTAAATAACGAAGAAGAAGGC
>dmgsat-10_D.leontia
AAAAAAGAAGGAGTAGAAGACTAAATAACGAAGAAGAAGGC
>dmgsat-11_D.bocki
TGTAAAAACTAATATAAATACAAATTCTGAAGTGGATACATGCATTATTAGCTGAGTTGTGAACTTTTTGTTTACAAATTTACAATTGAGTATGCAAATGTATTAGCCT
>dmgsat-11_D.leontia
TGTAAAAACTAATATAAATACAAATTCTGAAGTGGATACATGCATTATTAGCTGAGTTGTGAACTTTTTGTTTACAAATTTACAATTGAGTATGCAAATGTATTAGCCA
>dmgsat-12_D.bocki
TGGTGCTGCTGCCAGCGGCATTTCGGCTGCAGAGTCCTCGTCGGTCGTCGATAAGGGAGGACGGCTCGTCGACGGCCCTTCAGGTCGGCGTCGGGCGACGACGGCTGCGTAACC
TCCGGGCGGCTAGGTGCTGCTGCCGGTGACACCTCGGCTGCAGCGCAGCGTATCTGGGGCCCGGCCCGAAGATCTCGTCGGCAACTCCGCGCCGTCCTGGTCCGGTGAATCCCC
GAAGAGGTCGTCCATGGACCGGGGCCACTCGCTGATAAAGCGGCGGCGTGGACGTGCTGGGACGAGCGGTCTTCTGCCATCGTGGCGGATTCGTCGAGCAGGGGCCGGCGTCGG
TGGTGGATAACGGGCCGAGTGTTGCTCCGGACCGGCCAACTGGGGAGGCTCGGTGGATGACGAGCACAGCTCGTCGCACAGCTCCTCGGCTTTCCGTCGGGCAGCGGCGGCGGC
GTATTCGCCGGGGGTCGGCAGCGGAGGGCGGCT
>dmgsat-12_D.leontia
TGGTGCTGCTGCCAGCGGCATTTCGGCTGCAGAGTCCTCGTCGGTCGTCGATAAGGGAGGACGGCTCGTCGACGGCCCTTCAGGTCGGCGTCGGGCGACGACGGCTGCGTAACC
TCCGGGCGGCTAGGTGCTGCTGCCGGTGACACCTCGGCTGCAGCGCAGCGTATCTGGGGCCCGGCCCGAAGATCTCCTCGGCAACTCCGCGCCGTCCTGGTCCGGTGAATCCCC
GAAGAGGTCGTCCATGGACCGGGGCCACTCGCTGATAAAGCGGCGGCGTGGACGTGCTGGGACGAGCGGTCTTCTGCCATCGTGGCGGATTCGTCGAGCAGGGGCCGGCGTCGG
TGGTCGATTACGGGCCGAGTGTTGCTCCGGACCGGCCAACTGGGGAGGCTCGGTGGATGACGAGCACAGCTCGTCGCACAGCTCCTCGGCTTTCCGTCGGGCAGCGGCGGCGGC
GTATTCGCCGGGGGTCGGCAGCGGAGGGCGGCT
>dmgsat-13_D.seguyi
TATTTAGTTATTTAGT
>dmgsat-13_D.vulcana
TATTTAGTTATTTAGT
>dmgsat-13_D.mayri
TATTTAGT
>dmgsat-13_D.bakoue
TATTTAGTTATTTAGT
>dmgsat-13_D.pectinifera
TATCTAGT
>dmgsat-14_variant_1_D.nikananu
AAACTCAACCGATTTCAGCCTGGTTTCGTTTTTTCTTCTTAGTTTTTATAGGAGAACATTTCTGCATTCAAAACTTGATTTCCAATTTTTTGTCAATTTTGAAAAATTATTATG
ATGAAACCCCTTTTCTTTTTTTTAGTGCCTTAAAAATCGCTCCCCTTTTCTTTAATTTTTTAATCATAACTTGGCTT
>dmgsat-14_variant_2_D.nikananu
AAACTCAACCGATTTGAGTCTGGATTCGTTTTTTCTTCTTAGCTTTAATAGAAGAACTTTTCTGCATTTAAAACTCGATTTCTCATTTTTTGCCAAGTTTTGTAAAAAATGATG
AGAAAACCCCTTGCAATTTGTTTTTTAAATAGGTCATATTTTTGGCCTAAAAATCGCTAAAACCTCCAAGGGATTTTGATCATAACTTGGCTT
>dmgsat-14_variant_3_D.nikananu
AAACTCAACCGATTTCAGTCTGGTTTGGTTTTTTCTTCTTGGTTTTTAATGGAGAACATTTCTGCATTTAAAACTCGATTTCTAATTTTTGGCCAATTTTTGAGAATTTTGACG
ATGTAACCCCTTTAATTTTTTTTGGCTCAAAAATCGCGAACTTCTTCTGTGGTTTTTTGATCGTAACTTGGCTT
>dmgsat-14_D.jambulina
AAACTCAACCGATTTCAGTCTGGTTTGCTGTTTTCTTCTTGGTTTTTAAATGCGAACATTTCTGCATTTAAAACTCGATTTCTAATTTTTGGCCAATTTTTGACAATTTTAACG
ATGTAACCCCTTACATTTTTTTCAAAAATTGGGTCAAATTTTTTGGCTGAAAAATACTAAAACCCTCATTGGTTTTATGATCATAACTTGGCTA
>dmgsat-14_D.seguyi

AAACTCAACCGATTTCAGTCCGGTTTGGTTTTTTCTTCTTGGTTTTCAAAGGAGAACATTTCTGCATTCAAAAATTTAATTTCTAAATTTTTCCCAATTTTTTTAAATTTTGAC
GATGTAACCCCTTAAATTTTGTTTGTCCCAAAAATCGCATGGTTTTTTGATCATAACTTGGCTT
>dmgsat-15_D.jambulina
ATATGAAATATATGAAAT
>dmgsat-15_D.bunnanda
ATATGAAATATATGAAAT
>dmgsat-15_D.serrata
ATATGAAATATATGAAAT
>dmgsat-15_variant_1_D.seguyi
ATATGAAATATATGAAATATATGAAAT
>dmgsat-15_variant_2_D.seguyi
ATATGAA
>dmgsat-16_D.mayri
TGTATATTTGTATATT
>dmgsat-16_D.punjabiensis
TGTATATTTGTATATT
>dmgsat-16_D.bakoue
TGTATATTTGTATATT
>dmgsat-16_D.bocki
TGTATATTTGTATATT
>dmgsat-16_D.birchii
TGTATATT
>dmgsat-16_D.pectinifera
TATATAGTTA
>dmgsat-17_D.bakoue
TTATTATTGTTATTATTG
>dmgsat-17_D.punjabiensis
TTATTATTGTTATTATTG
>dmgsat-18_D.pectinifera
TAAATCGGCATTTTTATATCCTAATTTTTGGACAAATCAAACTTAAGAATAACCATAACTTGGCCAAAACAGCCTTAAAAACAGCGATTCGGAATATATATGGCATTTGGCTGT
CATTCCATTTTTGGCCAAATTATGCTCTTCAGAAGACATCAAATGCGGATTTTTGGGTCCT
>dmgsat-19_D.birchii
CATTTATTAGCCTATTTCATGAAATAGACCAATAATTCAAATTATTACACGAAATATTTTTTGATTATTATTGTTAACAAAAAATGTGTCTAGTTATTGAGGAAAATGTAATTT
AATACATAAAACAGGTTTAAATTACACAAATAAACC
>dmgsat-20_D.bunnanda
AATTTTAACGATGAGATCCCTTAAAAAACTTGAAAAAAATTTTAAAAATTTATTTTTCTTTTAAATATAGCTAGATCGATTCGCCTGTTGATTCTGATTAAGAATATATATGGA
TTACAGGGTCGGAAATAACTCCTTCACAGCGTTACAAGCTTCTGGGTTAAATTACAATATCATTCTTGATCGATATTTATCTATAATTTTATTGAACAATTGTTCAAAAAATCA
AAAAATAAAAATCTCGGAATTCGGAAAGCTTTTCTATGTTAGTTTTTCTTAGGTTAATAATTCTGCATACTAAATTTCAAATTTTTAAAAATTTAAATTTTTATCAATTTTTTT
TTTT
>dmgsat-21_D.serrata
GTAAAACTAAATATTTTACCGAGTTAAGCATACTTAATAAAACAAAACATCATTTGTACCGCGTTTTAAATATATATTTTGCGTTCGTCCAGCTTCTTAAATAAAATAGTTTAT
TTAAAGTAGCACCAAGTAATACTGTTGGGGCGCCCCTTAAGCATAAATTATATTGTTTTCCCAACCACGTATTCAATTTATAAAAATAAACAATGCCCAACTGAATAATATGAA
TATAACAACCCTCAATAATAAATCAAATTGCTTCTAAACCGA
>dmgsat-22_D.bunnanda

CAGTGAAGGAGTCATTTCCGACCCTATAAATCATATATATTCTTGATCAGCATCAACAGCCGAATCGATCTAGCCATCTCCGGACGAAAACTAAAATTTTCAAAAATTGGCCAA
AAATTGAATTTTTTTAAGTTTTAAACTGAGTATGCAGATTTATTAACCTACTAAAAACTAGCATAGAACAGCTTTCCGAATTAAAATTAATGCTTTTTTGGCTTAGTTATGAGA
TTTTTAAATCTTGATTTGTCGAAAAAATACTCATATAAAAATATCGAAAAATGTACTAAAAAATGGTATTATACTTTTATTCAGAAGCTTGTAACG

>dmgsat-22_D.serrata

CAGTGAAGGAGCCATTTCCGACCCTATAAATCATATATATTCTTGATCAGCATCAACAGGCGAATCTTTCTGGCCATCTCAGGTTGCAAAACAGACATTTTCAATTTTTTTCTAA
AAAATTAAAGGGGTTACATCATTAAAATTTGCAAAAAAAGGCCAAATCTTAACAAGTTAAATGGAGTATGCAGATTTGTTAAGCGCCAAACAACTAACACAGAACCGCTTTCCG
AATTGAAATTAATGCATTTTTGGCAAAGTTATGAGATTTTTAAACTTTGACTTTCCCAAAAAATACTAAAACAATTATGAAATAAAAATTTCGGTTTCAGGAATCGTAGAGGAA
AATATAATTTTTAT

>dmgsat-23_D.kanapiae

TGGTCTGATGACCTGATCTGGTAAACAGACC

>dmgsat-24_D.bunnanda

CATGGCTGCTGCATGGCTGCTG

>dmgsat-25_D.seguyi

CAAGTGTGACTTTTCATCCTCAATAGCCCATTTCCGGTCCCTTTCACGTGGCCATGGGCGGCAATGCGACGGGAGGCGTACGGTGCTCTCAACTGATCGGTCTCCTTCCTGACC
ATGTGTCTCACAGTGGCTACTTCCTGTTGAAACTAGTGGTTACTAAAGATAAAGCAAATTTGTGGCTAAGTATATTTACCACATTGACGACTGCCTTGCGGGGCGCTGTCTAGA
TGGGAGCTGCCTAAGTAGGCGTACCTCACCCGATCACCGTCGGACGAGGAGTGAAATTCCAAGGCTCTGGCCCGCCTTATATAGGGGCCTGACGATATTTAGTGTGCCCGCCGC
CTTTAAGAAATTTGTCATATTAGAGTGGCCAACCCCATTTTT

>dmgsat-26_D.nikananu

ATTATTAACACGGATGTGTACGGGTGAAAAGCTGCGGAGAGACTGAGGCTGCTGCGGCGAGTCGCCTCTATTTATAGGTATGAAAGATGGAGAGATTATATAAATAAATAGATT
TTTTGCGCTTACCGATCGGTCCTTTCAGCTTTATATATAGGATATGTATATGATAATAGTCTTGGTTCGAACTTACCCGTATTAGAGGACACTAGTTACCTTTCGTTGCAACAC
CGCAGACTTGGCCGAGATTCATCGATGATTAAGTCCTTTTGAGTGCAGAGGTTCTTTACCCAGTTATTAACACACTTGTTTACACACTTTTTAACACGTG

>dmgsat-27_D.truncata

TGATATGTACTTAAGCTCAGCTGCTAACTTAAGAACGACCATGGAAATCCTGGATTGTGACGTCATAAGATCGCCCTTCTCTGCGCTTCTCA

>dmgsat-28_D.pectinifera

CAACCAGTATCCAGGAATCTCCTCCACCAACAATTAGGTGCCAACATTATCACTGTTTAGCATGACCCAGTTCAGGATACACATTTTCCCGCAAAACGAGTGGCCAATCCCGGC
CAGAATAGAGCCAATCCTCTCCAGAATCGCATATTGGGGAAAGATGAGCATAGCAATGCATCCAAAAATGTACAATTGCCACCCATCTTATTTGGAGCCACCTTAAGAGCCTGG
GACTTGTCCTTTAGCATACACCTAGCTGCCACAATTTCACCGATTTAATGGGGGAGAGTCCATGAACAATCATTGCCAACGATATAGAGCCAATTATGGACCAATCCTCTCCAG
AATTCCACCTGATCCCGGCCAGAAATTGGCCAAGTATCAACAAACTCGCCAGTTGCCGGTCTCATTTACAGCCCTCACTCCTCCATGCATGACCCTGGCCAATAAACATGACCC
GCCATGGGAGCAGTGGCCAATCCTTTCCAGAATTTACCACAATTTCCTCAGCATTTCTAGCAAAATTGGGAGAAATTACCATTGCATATTATAACCTTCAATCCATATCGGCGG
AGGAGGGTCCATTATCCCTCAGAAGCGACCAGTTCCAGCCATCCAAGGCCTAATCCTCTCCAGAAACAGCCGATCCCGGCCAGAAATA

>dmgsat-29_D.bunnanda

ATCACCGAGCTATCACA

>dmgsat-30_D.kanapiae

ATTTAAGGCTTTTTACTGCCTTTGCACTTAAGCGCCTTTTAGTCGACAGCAGTTTTTAGTTTTTATTTACAAATAATATGAAAGCTAGGTGCGCCAGCATTGTGTAAAATGTTT
TTTACCTAAAAAGCTTTTCCAATATGGTCATATAAATTGAAAAAGTAAAATATAGCTGCGCCATCTATAGTGTAATTTTTCTTACAATGTATATTTTAAGTAACGTCAAATGTG
ATTTAACTAATACTAACAGCTAGCTGCGCCATCTATTGTGTAAAATGTGTGTATCATGTAAAATGTAGCAAGTACACATTATATTTCTTGACAATATGACAGTAGCGTTTAGGT
AGTGGTCTGGAGCTGATGTATACGCGTTCGGCTCCCGCGATCATACGACAAAATTGGTCAACAACGTATAATGGACACATGTGTATTGTGACAAGTATATCATTGTGACATGGG
ATCAATGGCAGAAGATAGGTGCAGTGGTAAATACTTTACCCACAGCATAGCGGGAAAGTGAAATAGTGGAGTGGTGTGGTTTTAGAAGTTCCGCGGGTGTGGCCGAGTGGGAGA
TATGCCAAAATGTGCGCAACACAGTCATTTTAAAGTATGCATGTGTGTTTTATACCAAATAAAGCTAGATAGTAAAAATATAAAATTATATTTAATCTTTTTATTGCAGGTTGA
GTATGCAACTTACAAAGTTTGTTTTCACGGTGGACAGTAGAGAAACAACAATAAAATATAGTGAAAATAAACGCAAATATGAGACAGCGGCAGCATTTTGGTAATATTTTTTTA
CA

>dmgsat-31_D.burlai

GACGCCATTTTGTCCCAATATTGCAACACAGCGATGGCGGTAGTAA

>dmgsat-32_D.bunnanda

AAAAAGCGCCACTAATTCGGGGTTTTGAACTATTATGTGGCGCCAATTCGCGGGCACATACGGCAACTATACATTTTATACGTGTAAAACCTATTTTACTACGTAAATCAAAAA

ATATCAAAAACGCGCCACTAATATGGTTTTTTGAACTATTATGTGGCTCCATTTCGCGGCCGCATACGGTAAACATACAATTTATACGTGTAGAGCATATTTTACCATAATTTT
CCTAATTTTGCTAAAATTGCCAAAATTCAAAAATTCTCTTATTATTAGCATTGCTCAGCGTCGTAAAGCTGATAAAACCGCTAGAAATCAAAAAATACATACAATTTATACGTG
TAAAACATATTTTCCTATGTATTTCTTAATTTTGCTAAAATGGCCAAAATCACAACATTTATATTATTATTAGCATTGCTCAGCGTCCTAAAGCTCATAAAACTGTTAGAAACA
CAAAAAATCGAAAAAGCGCCACTTTTTGGTTTTTTGAACTATTACGTGGCGCCAAGTCGCGGCCGCATACGGCAACTATACAATTTACACGTGTAAAACGTATTTACTACGTAA
ATCAAAAAATC
>dmgsat-33_D.pectinifera
ATACAAACACATGTAGAAAAACAT
>dmgsat-34_D.jambulina
GAATAGAGGA
>dmgsat-35_D.triauraria
TTAT
>dmgsat-36_D.watanabei
TGATATGATATGATATGATA
>dmgsat-37_D.pectinifera
TGTGATATTGTGATATATGTGATATT
>dmgsat-38_D.birchii
AATTTCAAAAATTTCAAA
>dmgsat-39_D.serrata
AGATATAGATATAGATAT
>dmgsat-40_D.bakoue
TGATATATTGATATAT
>dmgsat-41_D.bocki
CCCCCAATATTTCTTTAAAAAATTTTTAAAAATTTTGGGTAAATATAAAGGCAAAACTGAGTACGCAGTTTTGTTGAGCGTAGAAATCCCAATTCAGAACAGCTTTCCGCATTG
AATTTCATGCGTTTTTGGCCGAGTTATGAATTTTCTAAAATGGTTATTTCCCCCGAAAAATTATCACAATAAATGGCACCCATTTTTT
>dmgsat-42_D.vulcana
GAAGGAACAAGGAACAAGGAAGAAGGAA
>dmgsat-43_D.watanabei
GTTTGATTTGTTTGATTTGTTTGATTTATTTGATTTATTTGATTTATTTGATTTCTTTGATTTCTTTGATTTCTTTGATTT
>dmgsat-44_D.birchii
GAAAAGATTT
>dmgsat-45_D.truncata
TGAAAA
>dmgsat-46_D.jambulina
TACTTAATACTTACTACTTAC
>dmgsat-47_D.burlai
AATATATC
>dmgsat-48_D.bakoue
CTGT
>dmgsat-49_D.tani
GTTCCTTGGAGACGGCGATGACGATGATGTGGCCGTGGCGGCCCTTGGAGCCGATGGCGACGACGATGCGGCCGTGGTGGCCCTTGGAGACGGCAGCGACGATGATGTGGCCGT
GGTGGCCCTTGGCGACGATGACGATGGCGACGAGGATGCACCCGTGGTGGTTCTTGATGACGATGACGTGGCCCGAGAGCCCGATGCTGTGGTGACCGGTCT
>dmgsat-50_D.jambulina
ATAAATGATAAATGATAAATG
>dmgsat-51_D.burlai

ACGAAAAATACGAAAAAT
>dmgsat-52_D.bakoue
GTTTATT
>dmgsat-53_D.seguyi
ACAATAAAT
>dmgsat-54_D.pectinifera
TGTGATAGATAT
>dmgsat-55_D.truncata
GTTCAAAAATTTTGTCCAAAAATTTT
>dmgsat-56_D.bunnanda
CAACATCATCATCAG
>dmgsat-57_D.pectinifera
AAGCATGAAAGCATGA
>dmgsat-58_D.mayri
AAAATTTTGAAAAAATTG
>dmgsat-59_D.bunnanda
TTACGTTACACTACACTACA
>dmgsat-60_D.bocki
TTTTGAAAAAAATTTAAAAAA
>dmgsat-61_D.burlai
ATTCCACCCCTTAGTGTGACCGTATTGGCAACTTTGTTAGGTGTGACCATATTGGCAAAAGTCGTATTTGTCTATCGATTAAGATCTGGCGTTGCCAGACTTTTCGAACGCAAG
CCGATGGTGCTGCCAGACTTTTAACTTTTGAGGGATTTCGGCGTCTTTGTGTGCCCATCCGCCGTGGAGGCGGAAATGTTTTTGTTGTATTGCCAATGTTGTTGTAGGCAAAAA
CAAGGTCTTAATACCGCCTTTTAGCAGTACAAAATCATCAAATGCTGGATTTCATGCACCAGGCGCGCAGAAGCAGCAGCAAGTAGATAATAAAAAA
>dmgsat-62_D.mayri
GTTAAATAGTTAAATA
>dmgsat-63_D.burlai
TTCCCTACATGGGATTTGGACGTCGCCCAAATTGGCCAAAATTTGACTAAAAAATTGGGCCACGTATTTGAGCCTGGGACATCAACTAGATAAAGGTCCTAACTATTAGTATGC
TTAAAACTATACTTGTAACTGCAAGGATGCCAAAGTTAAGTAAATTCACGGAAGGGTCTGCTGCTGATGGCTGGCGGCAGCGCAGTCGGCGTCAATAAAAAATGACCTCTACCA
AAAAGGGTCGAAATTGGCCTATAAATTCCTGCCACGATCTCGGACTTGATGGTGTCCACAAGAGGATGGTCTGAGCTACCTGTGTGCCTAGTTTGAGATATATAGCTGCATGGA
TGAAGGAGTTATGCGATCCTGCCAATTGACGATTTCTGACGAAATTTTTTCCTCTCATCGTAATAATTCCAATCAAATCCGTTAAAATCCAAATGTAAATTGACGATGCGGAGA
AATGTAAACAAAACGTGCACTTACCCTGGAAAAGAGAAGAAAATATTAATAAATATTCTAAAGACTGATTATATTTAAATTACTCACCTTTGCATATAGATGTAACCGGATGGT
GATGATAAACAAACGTTGTTGGCTGCAATTTCCGGGTATTATTGCCTGCTTGCCCTTTGGAAATAAAATATATCATTATTAGCTAAATGAATTAATTAAATGTTTTAACAAATA
TTTTAATAAATGTCAAATGTCTGTCATATAATTATCATAAAATTAACAATAACTTAAATAAATAATATAAATAATCTTAATTATTAAATAGCGACGTGGGATAAAACCAGCAAG
CAGTCAATAAATAAACAACAATAATAAAAAACCAACTCATTAATATCTTGCATACAAAACGACATATTAAAAGATCCAATAAATTATTAAAAATATTATAAAAACTTAAATATT
TAAAAATTAAAATATTTAAAACGCATTTAAATTGGTAGACTTAGTACGGAGGTCGCCAAAAACTATCAAAAGGGGGATTAAAAACTTGGATACACATCTGGGGTCAATCATAGC
CAATAGATAAAGGCCTTGGCTAGTTGGTTGCTTAAAATGAGGTCCGTAACTCCAAGGATGCCGAAGGTATGCATTTGTTGCAATTGGAATCGATTATGCTGGCTGTTGTAAGCG
CCGTCCGCGCTAAGTTTTTGATGGTTTCCAAAAAAGGTCAAAACACAAAGAATTTCTTGGACCACGCCCTTCTCACCTTTTGGGTTGCCATTAGTAAGGTCTAAGCTAACTATG
TACACAAACTCAGGTCACTGGCTTCGAGGATGAGGGAGATCTCACTTTGCGTGAATTGTCAAAAACCGCACATATGCACGATCCGTGCGTCAGTATGGAGGCCCCCAAAATCGG
CGAAAATCTGACTTAAACACGCTTTTTACGCTATTCTGACAAGATGGTCGCCATGAGGGAGGTTCAAGCTAGTTATGAACAAAAAATTCAGGTCCCTAGCCCCAAGGACGAAGG
AGATCTCACTTTGCGCAAATTGGGTAGACAATCACACAAATGCACCGTCCGTGCGTGTGTATGGAGGTCGCCAAAAATGGCCAAAAAAGGCTTAAAAAACCGTCCACGCAATCG
GGCCTGGGGCTACTATTTGATAAAGGTCCAATCTAATTGTATGCAAAAAAGTAGTCCTGTAACTGCAAGGATGCCTGTTATGACAATCTACAGGTCTTGCTGCCCGTATATGAC
GGCAGTCTGTGCCGTAGTACAAAAATTACAAAAAATGGTCTTCGCCAAAAATGGCCAAAAAACGGACAAGTTTTTGGGCCACGCCTTCGAGCCTTTTTGGGTTGCCATTAGGAT
GGACTAAACTAACTGGCTACATAAATTCAGACCACTAGCTCTAAGGACATTGGAGAAATCTTGACGCGTAGCA
>dmgsat-64_D.seguyi

ACAAATA
>dmgsat-65_D.pectinifera
ATATCACCAATATCACCA
>dmgsat-66_D.bakoue
TCTTGATATCTTGTTA
>dmgsat-67_D.bunnanda
TGAAATCGGTTGAGTTTAAGCAAAGTTATGATGAAAAAACTATTTCATATGAAGCTCGATTTTTTCATTTTTTGATAAGGGGTTACATCATTAAAATTGTCAAAAATTGGAAAT
CGAGATTTGAATGCAGAAATGATCTCCTATAAAAACCAAGAAGAACACACCAAACCGAAC
>dmgsat-68_D.seguyi
CAACAACAG
>dmgsat-69_D.mayri
AAGTATATAAGTTTAT
>dmgsat-70_D.mayri
AAAACTGTCCAAAAACTATCGC
>dmgsat-71_D.bakoue
TGATATGG
>dmgsat-72_D.mayri
TGTTGCAGCCTGCCAATCTACGAAATTTGTGTAGCACAGAATTGGCGCTCTTCGACTGCACCTCCGTCACAAATATTTTTCTTGTTGTTCTAGCAGTCAAATTCTGTGTCTCAC
TGCTTTACCGCTATATAGTTGTTGCTGTTATTGCAGCCTACCTAATCTACGACCTCTGTGTTGCTCAGAATTGGCGGTCTTCGACTGCGACTCGCTCGCCGATGTCATGCTTGC
TGTTCTAGCAGTCGACCTCTGTGTCGCACTGCTTTAGCGCTCGCTTGTTGTTGCTGT
>dmgsat-73_D.bakoue
TCTTTATTCTTTAT
>dmgsat-74_D.mayri
ATGATAAA
>dmgsat-75_D.seguyi
TATTTGTACATTTGTA
>dmgsat-76_D.mayri
GTAGACTTGCGAGTCCGCCTCTCTCTGAAGACTTTCGAGTCCGCAAACT
>dmgsat-77_D.seguyi
TGATTCTCTTGCTCTTGCGGCTGACCTAGCGGTCCCCAAG
>dmgsat-78_D.seguyi
ATAAGTAAATAAGTAA
>dmgsat-79_D.bocki
TACCTATAATTTATAAGAAATTGGCGGAACAACAATCGGCGGAAAATCTCATAGCTCAGCCAAAAATGCATGAAATCCAATTCGGAAAGCTGTTCTGAATAGGTTTTTCTAAGC
TTAACAAAACTACATACTCAGTTTTGCTAAAATACTTACCTATAATTTATAAAAAATATTTAACCTATTGTT
>dmgsat-80_D.mayri
GTTTTAAAAATAAATCAAAATCAGCAGCAATTCAATTATCATAGTACACAGAGTCAGAGAAAGAGAGTGCGGGAGAGCGCAGTCTGTTAGTTCAATGGGGTCATAAAATTGGTT
TTAGCTGTGCGTTGATTCTTAGTTTTTAATAAAGGGTTAGTTCAATGCTTGTGTATAATTTGAATGAATTATTATTTCGTCTTTAGGTTCATGTATTTTTTTTTAATTTACCTA
GAGCTTCAGGAAAACCACCATATGAATCCTTAAAGTATCTGGATTCCCTCGAAAAAATTTCTTATGGCTTATTGACTTAAATCAGTGTTTTTTACTGTCAGAAGAAAATGGGGA
AGTTTTACAGATTTTTCAAAAGCGATAAAAATAGTAATTTTTTAATTTATTGTTAAGTTTAATTTTATTTTAAATTTTAA
>dmgsat-81_D.leontia
TGAACCTGGCGCCTCCACCGGAGCCGGGCACCTCCGCCGGAGCCGGACGCCACCGCGGGAACCGGACGCCTCCACCGGATCCAGGCGCCACCGCGGGAAACGGGCAGGACCGCG
TGAGCCGGGCGCTTTCATCAATTTCTTATAAATTATAGGTAAACAATAGGTTAAAATTTTTAAATAAATTATAGGTAAGTATTTTAGCAAAACTGAGTATTCAGAACAGCTTTC
CGAATTGGATTTCATGCATTTTTGGCTGAGCTATGAGATTTTCCGGCGATTTTTTCCCGCCATTTTTTTATAAATATAGGTAAAAAATCCGAGCGACCAAAAGTACCAGATCGT

CAGAAATATATATCGGCAAAAAATTCCAGAAATTAGTTGGCTATGAAAATATCGATACTTGGTATTTCTCAAATATATTCTCTTGGTCACACTAAGCCTCGCCTCGGCCCTATA
AAAGACGGGCCACAGCAGTGGGGAAGCCATTCGCCTGTTGACCGGCGATCGGTCAAGTACTCCGAGGGAGGAGTAACCAAGGAGTCATCCCGTGGAGTGGATCGCCGGATCAAT
GCGTCCATGTCCCGGGCACCACCGCGGGAGCCGGGCGCTACCACCGGAGCCGGTCACCTCTGACGAAACCGGGCGCCACCGCG
>dmgsat-82_D.nikananu
TTTCTTTGCTCGTTTTCTTTTTTTCTTTTGTCTCTTCTAGTTCTCCCTCTTCCTTTTGTCTCTCTTTCTTTCGCTCTCTTTCTTTTTTCTTTCTTCCTTTTGTCGCTCTTTCTT
CTTTCTTTCTCTCCGTCTTCCTTCTGTCTCTTCTTCTTTGGCTCCCTTTCTTCCTTTTGCCTTCCTTTCTTCTTTTTCTCTTCTTCCTTTTGTCTCTCTTTTAGTTCTCTCTTC
CTTTTTCTCTC
>dmgsat-83_D.seguyi
TACTTTTTGATTTTCAAAAATGTTTGAAAATTTTCGAAGTTTCATATTTTCGAAACGGGACGTGGTAAAAAAAAAATGAAAGAAGGGCAAACATGGTCAAACGAAGGGTAAAGA
TGGTCAACTTTCAAATTTCAAAAATTCAAATTTTGATAGAACACTTTTGAATATTTTCTGAATTTGGAAAATTTTCAAAATTCTAAAGGGTGGGCAAACGTGGTCAAACAATTC
TAATGCGATTTCCAAAAAAAAAATTTTGAAAAAAGTT
>dmgsat-84_D.mayri
TTTTTTGGGAATTTCAATTATCATAACTTGGCCAACAGGAAACTAATTTTGAATCGGAAAGCTGTTCTATGCTAGTTTTCAAAAGGCTAACAAATCTGCATACTTACTTTTTCA
ATAAACAAACGATTTAATTTTTTTGAATTTTGACGATGCAACCCCTTACAAAATTTGAAAAATTTTTTTGGCTCA
>dmgsat-85_D.burlai
AAAAAAAAAACGAACAAAAATTCAACTTTTGAAATCTGGCATTTAAGTATGCAATTTCATAGATACAGAAAGAAATAGCACAGAAAAGCTGTCGGAATCAAAATTAAAGCATTT
TTGACGGAATTGTAACTCCTC
>dmgsat-86_D.kanapiae
GACAAAATTATATCTAACATGCCATCAGAGATGCTCATCTAGCTATATTGCATTATTGG
>dmgsat-87_D.pectinifera
TATCACCAATATATCACGAATA
>dmgsat-88_D.birchii
AGATTTCACA
>dmgsat-89_D.jambulina
TCGTTACAGGCGACGGTCACACTGAACTTCGCCAGGGCCCTATATAAGGCGGGCGACACTCTTGGAATTTCACTCGGCGTCCGACAGCGATCCGATGAGGTAGCTTATATCCAG
AGAACAACCCGCCAGGCAGCTGCCAGTGTGGTGAATATACTAAGCAACAAATTTGCTTGATTTGTAGTAACTAATACTTACTCAACAGGAAGTAGCTACCCTGAGGCACATGTT
CAGGATGAAGGCCGCAGGATTGAGAGCACCCTGCACCTCCTGTCGCATAACCGCGCACAGCCCACAGTATAGGAAAAATAACATAAAGAATTCGAGTCATAAGAAAATATTTGA
ATTTAATGCGCGAGCGCAATTGATCGTAAACGCCAGGTCACACTGGATCGAACAGGCGTTGGCCACACTAATACGGCAATT
>dmgsat-90_D.vulcana
GTGAAAAGACTACCCATGATAGGGTTG
>dmgsat-91_D.punjabiensis
GAAGCCTTTCTCTCTAACGGTCTGGCAACCCTGAAAATATGGATGAGAATCGCAGAGAAGGGCGCTTCACCTTAACAGTATGTAATTTGCAGAGGAAGGCCTACCTTAAGTACA
CAGTATGAGAATCGCAGAGAATTTGCGTTGCAAACTTCTGCCTGAAATTATAATACCCTGCAAGGGTATAAAAAGTGTAACCCCGAGTTAAGTATAGAAAGCTAATATTTTTAT
GGCTGCCATATTTCATTTGATTAAAGTCTATCAACCGCCAAACATTAGATTAGTTGGTTTTAAACAAAGTTTGGAGTTTCTAGCCTTGACAACATCTATATGTGAATATCTATG
CAAAAAGAAAATTATACATAAAGAGGAAATGACAAGTGCAAAAAAGGGAATTGCTTAGGTATGTAAATCGAAAAGAGAGGCACTTACCAACAGAGTATGTAGAATCGCAGAGG
>dmgsat-92_D.burlai
TGGCGCGAGGACCGCCGTCTACCATTGACAATTTCTGGCCCAACATTGTGAGCGGATTGCATGGGATCGCAGCAGAAGATCCAGCTCATCCAGCAAGTGGTGACGCCCAAGGGC
GAGCTGACGAATGTCCCGGTGAGTGTAAGCAGGGATATATTCAACCGAAAGTCAAACTAACGAGCAACTTCTTTCCCTCAGATCGCCACCAATGCCAATGAAAAAAAATCTCGC
ACATGTGTGCATATAATTCTCAACTGGTTCTAACCTCCCCGGTATGAAGACACGGCCAAGTCGCAGGTGAACAATGTGATTTAAGGAGATTGTGGAGCGCGCCAAGCCGCTGTC
GATCCGCCTGACTAACCCCAACGATCGTCTGAGTGGTGGAACTGAGCTCGGCCGTCGCCCAGTCGTACGCCGCTGGCAAGGGCCAGGCTTCAACGCCGG
>dmgsat-93_D.tani
CATCAGATAGGAATAAATGTGGTGTCGTTTGCTCCGTATTTAACACCAGAAGACAACAGTACCTACATCGTTTCTGTTGCTTAAGCCTTTTAAAAGTTATGGGCAGATTATTTT
TTATTGAATATATTTTGAACCGAG
>dmgsat-94_D.kanapiae

TGAAAAATTTGAAAAATT
>dmgsat-95_D.punjabiensis
CAAGAAGAAGAACAAGGAGAAATGGTTCCCCGCCCTCGTTGTGACGCCTACTACACAGGTGAGCTGAGAATTTGTTTTCATACAGGATATAATAATGATTGATTGCTTTTCTAC
ACAGGCCACAGTCCGCATCCGCGTGAAGGACGAGTACCTGGTACATTCGTCCAAGGACGGCCGTTACTATATGGTCCCGAAGAAGGAGGCCACCGAGTACACCCGCGCAGTGGC
AAGTTGCGTTCACCGCCTCCCAGTCAGCGGTTGGAGTGGGAGCAGCAGCCCCCGGAGCAGTAGTTGCCTTGGTAATTACCGCTGTGTTGCCGCCAACAGCGGGTGCAGGATCAG
GCGCATCCGGAACGGGTTCGGCCACAGCAACGGCCACTACCTCAGGTGGCGCTGCTGTCGTGGTAAGCTCGGCGGCCAGGAAGCAGGCCCTCAAGGCAAGCGCCATTCAGCATA
GCCTAAAAGGACGACTGACGCCCTCGGCCGTGGCCAATCCAGTCAAGATGCACACGCCACGAGGAGCAGAAGCAGCGTCTGCCAAGGAGGTGGTCAACGAGAAGGAGAAGAATA
TCGGCAAGGTGGTGTGCGTGGAGACAGAGTC
>dmgsat-96_D.punjabiensis
CTTATTCTCAATCTCAATCTGAATCTCATT
>dmgsat-97_D.bunnanda
TTAAAATTTAAATTTTTTG
>dmgsat-98_D.bunnanda
GAATGAGGAGGAATGAGGAG
>dmgsat-99_D.truncata
AAATATACCA
>dmgsat-100_D.seguyi
GAAAGATGAT
>dmgsat-101_D.bunnanda
ACAATGTAACAACACAATA

Figure S1. Graph layouts of dmgsat-1 clusters retrieved by TAREAN. (A). *D. asahinai* dmgsat-1 cluster. **(B)** *D. tani* dmgsat-1 cluster. **(C)**. *D. lacteicornis* dmgsat-1 cluster. **(D)**. *D. rufa* dmgsat-1 cluster. **(E)**. *D. auraria* dmgsat-1 cluster. **(F)** *D. triauraria* dmgsat-1 cluster.

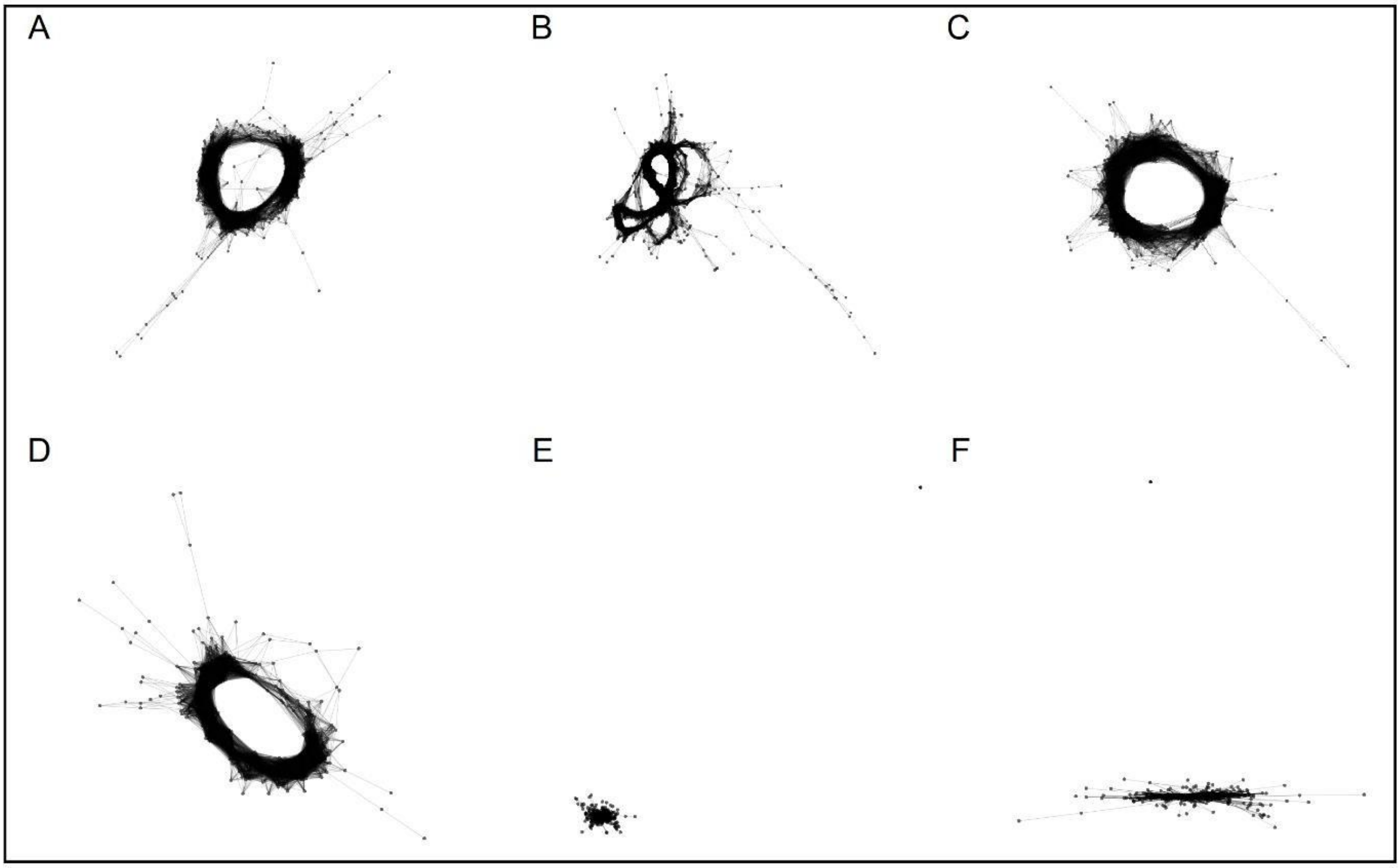

Figure S2: Correlation test between genome size and contribution to genome of the 397 initial clusters retrieved by TAREAN (see Figure 2) from the 23 analyzed species from the *montium* group. The p-value was obtained with Spearman's correlation test. The colors are based on subgroup classification proposed by Yassin (2018) [29].

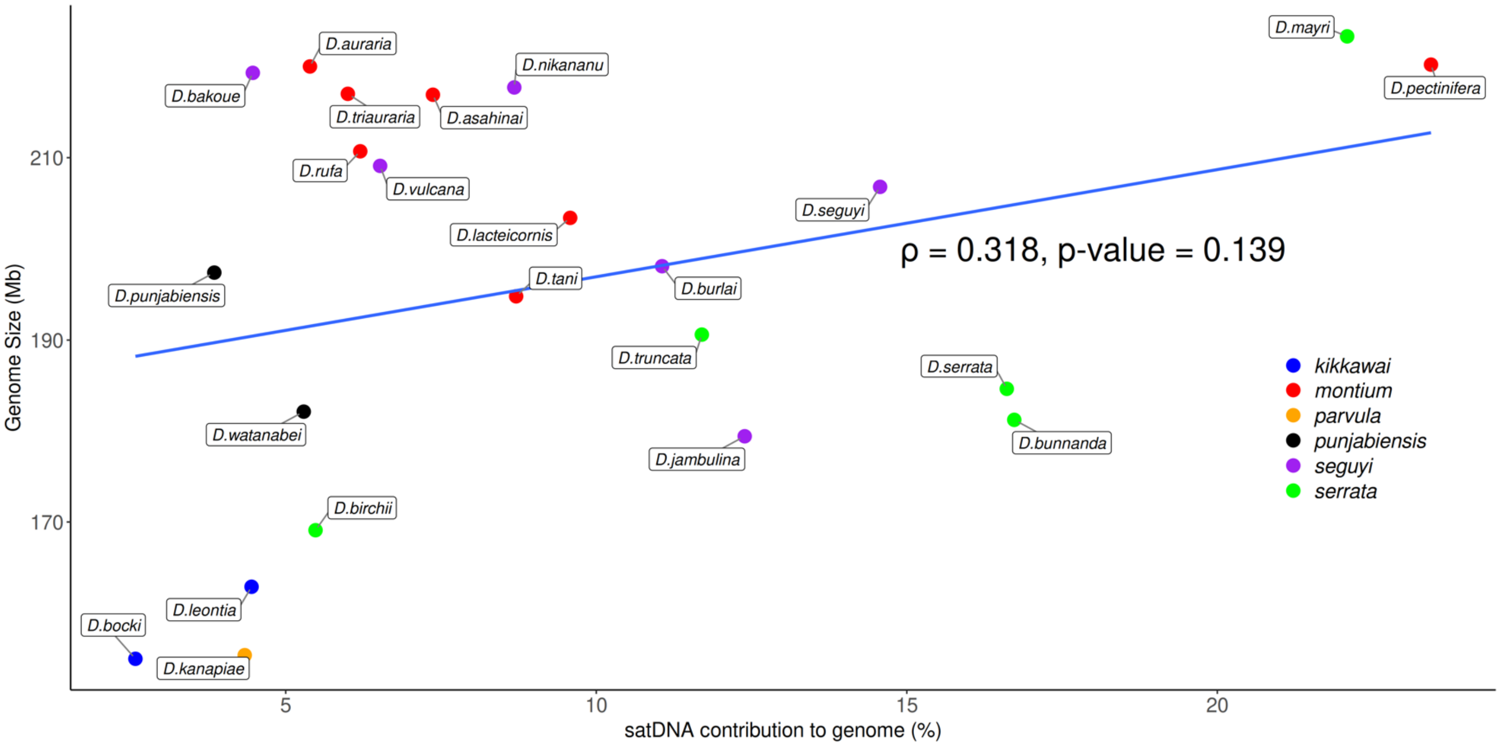

Table S2. SatDNA families in the *montium* group sharing homology with Helitron transposable elements.

SatDNA Family	Species	Consensus size (in bp)	Helitron Hit (s) (Repbase search)	SatDNA hit fragment (From/To) "bp"	Correspondent fragment on Helitron sequence (From/To) "bp"	Similarity value between 2 aligned fragments
dmgsat-1	*D. asahinai*	374	DNA4-1_DK#RC/Helitron	19-201	126-312	0.7845
dmgsat-1	*D. rufa*	368	DNA4-1_DK#RC/Helitron	23-212	117-312	0.7720
dmgsat-1	*D. lacteicornis*	374	DNA4-1_DK#RC/Helitron	134-316	126-312	0.7889
dmgsat-1	*D. tani*	374	DNA4-1_DK#RC/Helitron	50-230	137-320	0.7337
dmgsat-1	*D. auraria*	367	DNA4-1_DK#RC/Helitron	1-97	223-320	0.7959
dmgsat-1	*D. triauraria*	366	DNA4-1_DK#RC/Helitron	227-359	187-320	0.8000
dmgsat-7	*D. mayri*	150	Helitron-N4_DSer	25-150	435-562	0.7795
dmgsat-7	*D. serrata*	153	Helitron-N4_DSer	1-149	344-491	0.9195
dmgsat-8	*D. truncata*	198	DNA4-1_DK#RC/Helitron	49-191	174-315	0.7413
dmgsat-8	*D. truncata*	194	DNA4-1_DK#RC/Helitron	28-191	146-315	0.7305
dmgsat-14	*D. jambulina*	208	DNA4-1_DK#RC/Helitron;	24-190	138-313	0.7193
dmgsat-14	*D. seguyi*	178	DNA4-1_DK#RC/Helitron	13-178	132-313	0.7229
dmgsat-20	*D. bunnanda*	346	DNA4-1_DK#RC#Helitron	1-175<br>229-340	10-184<br>188-299	0.7543<br>0.7857
dmgsat-22	*D. bunnanda*	324	DNA4-1_DK#RC/Helitron	48-314	10-322	0.8303
dmgsat-22	*D. serrata*	356	DNA4-1_DK#RC/Helitron	21-291	42-322	0.8168
dmgsat-41	*D. bocki*	202	DNA4-1_DK#RC/Helitron	54-148	223-320	0.7396
dmgsat-67	*D. bunnanda*	174	DNA4-1_DK#RC/Helitron	56-107	131-193	0.9259
dmgsat-79	*D. bocki*	186	DNA4-1_DK#RC/Helitron	46-138	223-315	0.7634
dmgsat-81	*D. leontia*	653	DNA4-1_DK#RC/Helitron	216-267	264-315	0.8269
dmgsat-84	*D. mayri*	189	DNA4-1_DK#RC/Helitron	19-177	145-314	0.7546
dmgsat-91	*D. punjabiensis*	455	Helitron-N4_DSer	138-186	1-49	0.9796

Table S3. Top ten contigs (sorted by total score) containing copies of dmgsat-7 in *D. serrata.*

Species	NCBI Accession	Accession length (bp)	Largest number of uninterrupted repeats found
Drosophila serrata	MTTC01000695.1*	82,685	540
	MTTC01000697.1*	54,943	359
	MTTC01001044.1	93,247	428
	MTTC01000698.1*	44,750	292
	MTTC01000455.1	32,409	180
	MTTC01000722.1	44,489	229
	MTTC01000425.1	48,635	172
	MTTC01000872.1	59,640	162
	MTTC01000440.1	155,132	319
	MTTC01000910.1	164,193	138

* Contigs containing only CTR sequences
